# Exploring the mechanism of Buyang Huanwu Decoction in the treatment of spinal cord injury based on network pharmacology and molecular docking

**DOI:** 10.1097/MD.0000000000031023

**Published:** 2022-10-07

**Authors:** Ying Wang, Haixu Chen, Junwei Wang, Xin Chen, Lan Chen

**Affiliations:** a Department of Basic Medicine, Sichuan Vocational College of Health and Rehabilitation, Zigong, China; b Department of Pediatric Surgery and Vascular Surgery, Zigong Fourth People’s Hospital, Zigong, China; c Department of Integrated Traditional Chinese and Western Medicine for Pulmonary Disease, Zigong First People’s Hospital, Zigong, China.

**Keywords:** Buyang Huanwu Decoction, mechanism of action, molecular docking, network pharmacology, spinal cord injury

## Abstract

Buyang Huanwu Decoction, a traditional Chinese medicine decoction, is widely used to treat spinal cord injury in China. However, the underlying mechanism of this decoction in treating spinal cord injury is unclear. This study used network pharmacology and molecular docking to examine the pharmacological mechanism of Buyang Huanwu Decoction in prevention and treatment of spinal cord injury. The active compounds and target genes of Buyang Huanwu Decoction were collected from the Traditional Chinese Medicine Systems Pharmacology and the SwissTargetPrediction Database. The network diagram of ”traditional Chinese medicine compound target“ was constructed by Cytoscape software. Genetic data of spinal cord injury were obtained by GeneCards database. According to the intersection of Buyang Huanwu Decoction’s targets and disease targets, the core targets were searched. The protein-protein interaction network were constructed using the STRING and BisoGenet platforms. Meanwhile, gene ontology enrichment and Kyoto encyclopedia of genes, and genome pathway were performed on the intersection targets by Metascape. Molecular docking technology was adopted to verify the combination of main components and core targets. A total of 109 active compounds and 5440 prediction targets were screened from 7 Chinese herbal medicines of Buyang Huanwu Decoction, with 98 active components and 49 related prediction targets being strongly linked to Spinal Cord Injury. By studying protein-protein interaction network, a total of 8 core proteins were identified, primarily interleukin-6, tumor protein P53, epidermal growth factor receptor, and others. Positive regulation of kinase activity regulation of reaction to inorganic chemicals are the basic biological processes. Buyang Huanwu Decoction cures Spinal Cord Injury primarily by moderating immunological inflammation, apoptosis, and oxidative stress, which involves the cancer pathway, the HIF-1 signaling pathway, the p53 signaling pathway, the MAPK signaling pathway, and so on. The results of molecular docking demonstrated that the primary components could attach to the target protein effectively. Finally, the mechanism of Buyang Huanwu Decoction in the treatment of spinal cord injury through multicomponent, multitarget, and multichannel was deeply explored. And it offers new ideas and directions for future research on the mechanism of the treatment of spinal cord injury.

## 1. Introduction

Spinal cord injury (SCI) is a common and devastating neurological injury that can result in significant neurological damage and possibly paralysis around the world.^[1]^ The primary pathogenic mechanisms of SCI include axonal separation, neuronal death, and, eventually, lifelong neurological impairment.^[2]^ Acute spinal cord injury (ASCI) is caused by two mechanisms: primary and subsequent injury. The term “primary injury” refers to the first mechanical lesion to the spinal cord, whereas “ subsequent injury “ refers to a set of biochemical and cellular processes involved in SCI, such as oxygen free radical generation and the inflammatory response after trauma.^[3]^ The literature reports that the global incidence of SCI is 10.4 to 83 cases/million/year.^[4]^ According to the latest SCI epidemiological data in China, the incidence rate has risen quickly in the last decade, with the elderly (65–74 years old) having the greatest incidence rate, with an average annual incidence rate of 127.1 cases/million people.^[5]^ SCI can result in major consequences, in China, the proportion of quadriplegia and complete damage is as high as 37.4% to 82.0% and 14.1% to 73.9%, respectively.^[6]^ SCI will not only bring considerable bodily and psychological impairment to the sufferer, but will also impose a significant economic burden on society as a whole. As a result, its treatment is a critical issue.

The goal of SCI treatment is to promote spinal cord tissue regeneration and functional recovery. SCI treatment is currently separated into two stages: anti-inflammatory symptomatic treatment in the acute stage and rehabilitation function exercise in the latter stage.^[7]^ The latter is critical in the treatment of SCI patients, yet current physical and rehabilitative exercises are ineffective.^[8]^ Thus, finding a more effective treatment strategy is critical. SCI is classified as “low back pain,” “atrophy syndrome,” and “long bi” in traditional Chinese medicine (TCM). According to studies, TCM has obvious therapeutic effects on it, particularly when used later in the recovery process.^[9]^ Buyang Huanwu Decoction (BYHWD), which is composed of Astragalus membranaceus, Angelica tail, Red Peony Root, Earthworm, Sichuan lovase rhizome, Carthamus tinctorious, Persicae Semen, has been shown to improve the ischemic and inflammatory response of post-injury tissues to reduce post-injury tissue edema, inhibit lipid peroxidation, neural cell apoptosis.^[10–13]^ However, because TCM prescriptions have complicated components and targets, network pharmacology methodologies must be used to describe their distinct pharmacological mechanisms.^[14]^

Data mining may be fully utilized in network pharmacology to disclose the association between TCM chemicals and diseases, as well as to clarify the mechanism of TCM to treat diseases.^[15]^ Molecular docking may anticipate the link between the active component and the target protein based on receptor features, receptor interaction mode with the drug molecule, and intermolecular binding mode and affinity, making it more powerful in proving the drug’s mechanism of action.^[15,16]^ Finally, the objective of this study is to systematically reveal the internal mechanism of BYHWD in the treatment of SCI from multiple levels and perspectives using network pharmacology in conjunction with systems, bioinformatics, network science, and other disciplines, in order to provide better clinical guidance.

## 2. Materials and Methods

### 2.1. Collection and screening of active ingredients and targets of BYHWD

The TCM Systematic Pharmacology Database^[17]^ (TCMSP, http://lsp.nwu.edu.cn/tcmp.php) was used to collect the relevant chemical components of BYHWD -Chi Shao, Chuanxiong, Angelica, Di Long, and Huang Qi. To acquire the active ingredients of BYHWD, the active ingredients of each medicine were screened based on oral bioavailability (OB) $\underset{-}{\geq }$ 30% and drug-like characteristics (DL) $\underset{-}{\geq }$ 0.18, and then merged and de-weighted. To collect the action target ts of the components, the TCMSP database and SwissTargetPrediction database (http://www.swisstargetprediction.ch/) were used, and the Uniprot database (http://www.uniprot.org/) was used. “Protein names” were universally adjusted to official names.

### 2.2. Drug-component-target network construction and analysis

Import the active ingredients and predicted target information from BYHWD and “1.1” into Cytoscape 3.7.2 software^[18]^ to create a drug-component-predicted target network diagram, and then use Cytoscape 3.7.2 software’s Merge function to create the final drug-component-target network. The nodes represent TCMs, active substances, and targets, while the edges reflect the relationships between TCMs and ingredients, as well as between ingredients and targets. Analyze the node degree value of the aforementioned network using the software’s “ Networkanalyzer” plug-in. The greater the value, the more significant the node in the network. BYHWD appears to play a major role in the treatment of SCI based on the components with higher node degree values.

### 2.3. Screening and prediction of core targets of BYHWD in the treatment of SCI

To screen disease targets, use the human gene database GeneCards (https://www.genecards.org/) and enter keywords such as “spinal cord injury” to retrieve SCI-related targets. Upload the anticipated targets and SCI-related targets from “1.1” to the WeChat platform (http://www.bioinformatics.com.cn/), choose “Venn Diagram,” and get BYHWD in the treatment of potential SCI targets.

### 2.4. Construction and topology analysis of protein–protein interaction network

The protein-protein interaction (PPI) network of putative therapeutic targets of SCI was examined in this study utilizing the STRING database (https://stringdb.org). To obtain protein-protein interactions, the possible therapeutic targets were imported into the Search Tool for the Retrieval of Interaction Gene/Proteins (STRING) database, the species was set to human, and a moderate interaction value of “0.4” was used. The data was displayed and analyzed using Cytoscape 3.7.2 software, and the topological features of prospective treatment targets were investigated using Cytoscape’s cytoNCA function.

### 2.5. Gene ontology and Kyoto encyclopedia of genes and genomes enrichment analysis

The BYHWD junction genes in the therapy of SCI were imported into the biological information annotation database Metascape (https://metascape.org/), and the species was restricted to “H. sapiens.” For GO enrichment analysis of the targets of BYHWD in the treatment of SCI, the biological process (BP), cellular component (CC), and molecular function were chosen; pathway enrichment analysis of target genes was performed using the KEGG database. To screen out biologically transgenic target pathways with substantial differences, the threshold was set to *P* < .05, and the *P* value was ordered in increasing order. Create bar and bubble charts from the results.

### 2.6. Composition-target molecular docking

This study used AutoDockTools-1.5 to see if the beneficial chemicals in BYHWD could attach to target proteins in the body and exert their curative effect after entering the body. Molecular docking between the core compound and the core target can successfully determine the small molecule compound that matches the spatial and electrical properties of the target receptor’s active site, as well as whether the compound can bind to the target after entering the body. Check the precision of compound target predictions. Enter the name of the potential target protein into the PDB (http://www.rcsb.org/pdb/) database, find the PDBID of the corresponding protein, and save it in pdb format. Next, search the TCMSP database for the compound component corresponding to the target protein, download and save it as mol2 format, import the target protein data information and its corresponding active ingredient information into AutoDockTools-1.5.6 software, dewater and hydrogenate the protein, dock the small molecule following hydrogenation to acquire the docking binding energy of the drug and the target protein, and then do visual analysis with PyMOL-2.5.0 software.

## 3. Results

### 3.1. Prediction of compounds and active ingredients in BYHWD

The TCMSP database yielded 109 chemicals (Radix Paeoniae 29 + Chuanxiong 7 + Angelicae 2 + Dilong 6 + Safflower 22 + Astragalus 20 + Peach kernel 23) after deleting duplicate entries and using 0B $\underset{-}{\geq }$ 30% and DL $\underset{-}{\geq }$ 0.18 as screening criterion. Following screening, 98 active components (Radix Paeoniae 21 + Chuanxiong 6 + Angelicae 2 + Dilong 6 + Safflower 20 + Astragalus 20 + Peach kernel 23) were found, which are given in Table 1, and the primary active ingredients of B uyang Huan wu decoction are displayed in Table 2. After SwissTargetPrediction predicted a total of 5440 target proteins corresponding to the obtained active ingredients, the target proteins were standardized into 834 using the uniprot database Gene (Chishao 866 + Chuanxiong 423 + Angelica 82 + Dilong 523 + Honghua 1292 + Astragalus 1174 + Taoren 1080). According to the table, red peony root has the most medicinal benefit in BYHWD, followed by peach kernel and safflower. As can be observed, BYHWD primarily stimulates blood circulation and eliminates blood stasis, as well as treating qi shortage and blood stasis illness.

**Table 1:** 

Traditional Chinese Medicine	Compound/piece	Active ingredient/pc
red peony	29	21
Chuanxiong	7	6
Angelica	2	2
Earthworm	6	6
safflower	22	20
Astragalus	20	20
peach kernel	23	23
Total	109	98

**Table 2:** Main active ingredients in Buyang Huanwu Decoction.

Molecule ID	Molecule name	OB/%	DL	HL	Source	
MOL002714	Baicalein	33.52	0.21	16.25		red peony
MOL004355	Spinasterol	42.98	0.76	5.32		red peony
MOL006999	stigmast-7-en-3-ol	37.42	0.75	5.85		red peony
MOL007004	Albiflorin	30.25	0.77	7.83		red peony
MOL007025	Isobenzoylpaeoniflorin	31.14	0.54	21.1		red peony
MOL002883	Ethyloleate (NF)	32.4	0.19	4.85		red peony
MOL001494	Mandenol	42	0.19	5.39		Chuanxiong
MOL002135	Myricanone	40.6	0.51	4.39		Chuanxiong
MOL002151	Senkyunone	47.66	0.24	2.42		Chuanxiong
MOL002157	Wallichilide	42.31	0.71	6.85		Chuanxiong
MOL000359	Sitosterol	36.91	0.75	5.37		Chuanxiong
MOL000358	beta-sitosterol	36.91	0.75	5.36		Angelica
MOL000449	Stigmasterol	43.83	0.76	5.57		Angelica
MOL000953	CLR	37.87	0.68	4.52		Earthworm
MOL005030	gondoicacid	30.7	0.2	4.79		Earthworm
MOL006202	LAX	44.11	0.2	5.63		Earthworm
MOL008698	Dihydrocapsaicin	47.07	0.19	2.98		Earthworm
MOL010485	EPA	45.66	0.21	5.35		Earthworm
MOL005320	Arachidonate	45.57	0.2	7.56		Earthworm
MOL002712	6-Hydroxykaempferol	62.13	0.27	14.29		safflower
MOL002694	4-[(E)-4-(3,5-dimethoxy-4-oxo-1-cyclohexa-2,5-dienylidene)but -2-enylidene]-2,6-dimethoxycyclohexa-2,5-dien-1 -one	48.47	0.36	3.24		safflower
MOL002710	Pyrethrin II	48.36	0.35	1.79		safflower
MOL000098	Quercetin	46.43	0.28	14.4		safflower
MOL002757	7,8-dimethyl-1H-pyrimido[5,6- g]quinoxaline -2,4-dione	45.75	0.19	-0.72		safflower
MOL002721	Quercetagetin	45.01	0.31	13.82		safflower
MOL000422	Kaempferol	41.88	0.24	14.74		safflower
MOL000953	CLR	37.87	0.68	4.52		safflower
MOL000006	Luteolin	36.16	0.25	15.94		safflower
MOL002714	Baicalein	33.52	0.21	16.25		safflower
MOL002719	6-Hydroxynaringenin	33.23	0.24	15.67		safflower
MOL000211	Mairin	55.38	0.78	8.87		Astragalus
MOL000239	Jaranol	50.83	0.29	15.5		Astragalus
MOL000354	Isorhamnetin	49.6	0.31	14.34		Astragalus
MOL000371	3,9-di-O-methylnissolin	53.74	0.48	9		Astragalus
MOL000380	(6aR,11aR)-9,10-dimethoxy-6a,11a-dihydro-6H-benzofurano[3,2- c]chromen -3-ol	64.26	0.42	8.49		Astragalus
MOL000398	Isoflavone	109.99	0.3	15.51		Astragalus
MOL000422	Kaempferol	41.88	0.24	14.74		Astragalus
MOL000438	(3R)-3-(2-hydroxy-3,4-dimethoxyphenyl)chroman -7-ol	67.67	0.26	2.9		Astragalus
MOL000098	Quercetin	46.43	0.28	14.4		Astragalus
MOL001323	Sitosterolalpha1	43.28	0.78	5.64		peach kernel
MOL001328	2,3-didehydroGA70	63.29	0.5	7.62		peach kernel
MOL001329	2,3-didehydroGA77	88.08	0.53	7.6		peach kernel
MOL001339	GA119	76.36	0.49	8.35		peach kernel
MOL001340	GA120	84.85	0.45	8.4		peach kernel
MOL001342	GA121-isolactone	72.7	0.54	7.63		peach kernel
MOL001343	GA122	64.79	0.5	7.01		peach kernel
MOL001344	GA122-isolactone	88.11	0.54	7.4		peach kernel
MOL001350	GA30	61.72	0.54	8.74		peach kernel
MOL001352	GA54	64.21	0.53	10.19		peach kernel
MOL001353	GA60	93.17	0.53	7.9		peach kernel
MOL001358	gibberellin7	73.8	0.5	9.79		peach kernel
MOL00136 0	GA77	87.89	0.53	7.28		peach kernel
MOL001361	GA87	68.85	0.57	8.76		peach kernel
MOL001371	Populoside_qt	108.89	0.2	5.86		peach kernel
MOL000296	Hederagenin	36.91	0.75	5.35		peach kernel
MOL000358	beta-sitosterol	36.91	0.75	5.36		peach kernel

DL = drug-likeness, OB = oral bioavailability.

### 3.2. Drug-component-target network construction and topology analysis

**Figure 1.**
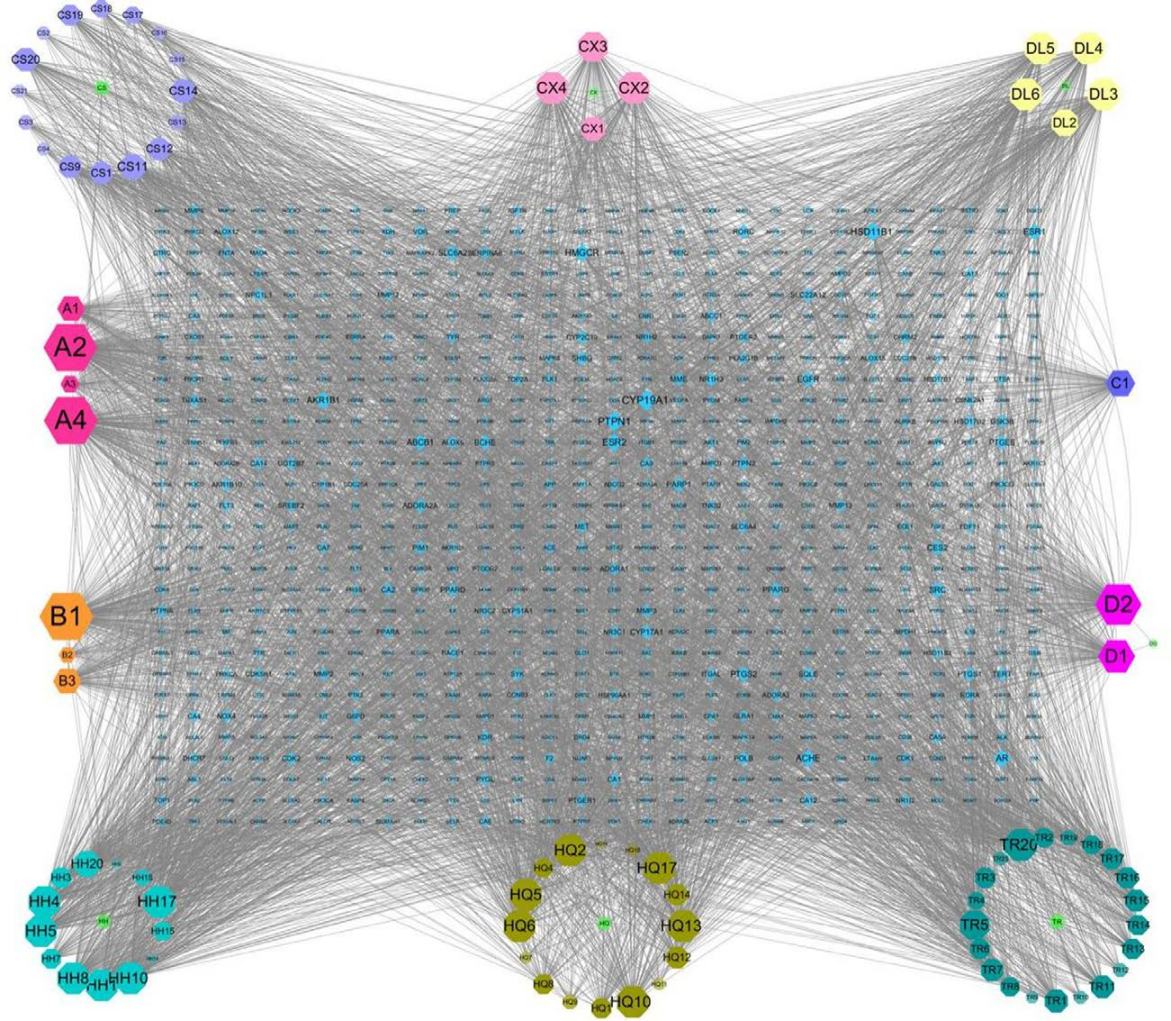
Interaction network of Buyang Huanwu Decoction.

To fully understand the molecular mechanism of BYHWD in the treatment of SCI, a drug-component-target network was built. The drug-component-target network was created and visualized using Cytoscape 3.7.2’s Merge function, as shown in Figure 1, where the green circle represents TCM, the colored octagon represents the active compound, and the central turquoise blue diamond represents the disease-drug common target. A total of 926 nodes were found, including 7 for TCM, 85 for active chemicals, and 834 for disease-drug common targets. Figure 1 shows that Tonic Yang Returning Five Soup exerts its therapeutic effect via multi-drug-multi-component-multi-target interactions. Then, using Cytoscape’s Network Analyzer tool, compute the important topological properties of network nodes, such as Degree value, Betweenness Centrality (BC), Closeness Centrality (CC), and Topological Coefficient. The core chemicals that were larger than or equal to the median Degree values were Hederagenin, Quercetin, Baicalein, Beta-Sitosterol, and Stigmasterol (Tables 3 and 4), all of which may be key components for the therapy of SCI.

**Table 3:** Topological parameters of core targets.

Ingredient name	Degree	BC	CC	TC
Hederagenin	202	0.016739	0.371934	0.169818
Quercetin	202	0.016134	0.371934	0.1655
Baicalein	202	0.031227	0.371635	0.162532
Beta-Sitosterol	168	0.004531	0.345278	0.241228
Stigmasterol	126	0.004326	0.342974	0.240606

BP = biological processes, CC = closeness centrality, TC = topological coefficient.

**Table 4:** Drug identical ingredient list.

MolID					Same ingredient code
MOL000296	Astragalus	peach kernel			A1
MOL000422	Astragalus	safflower			A2
MOL000433	Astragalus	Chuanxiong			A3
MOL000098	Astragalus	safflower			A4
MOL002714	red peony	safflower			B1
MOL002776	red peony	safflower			B2
MOL000359	red peony	Chuanxiong			B3
MOL000953	Earthworm	safflower			C1
MOL000449	Angelica	red peony	safflower		D1
MOL000358	Angelica	red peony	safflower	peach kernel	D2

### 3.3. The target of BYHWD in the treatment of SCI

**Figure 2.**
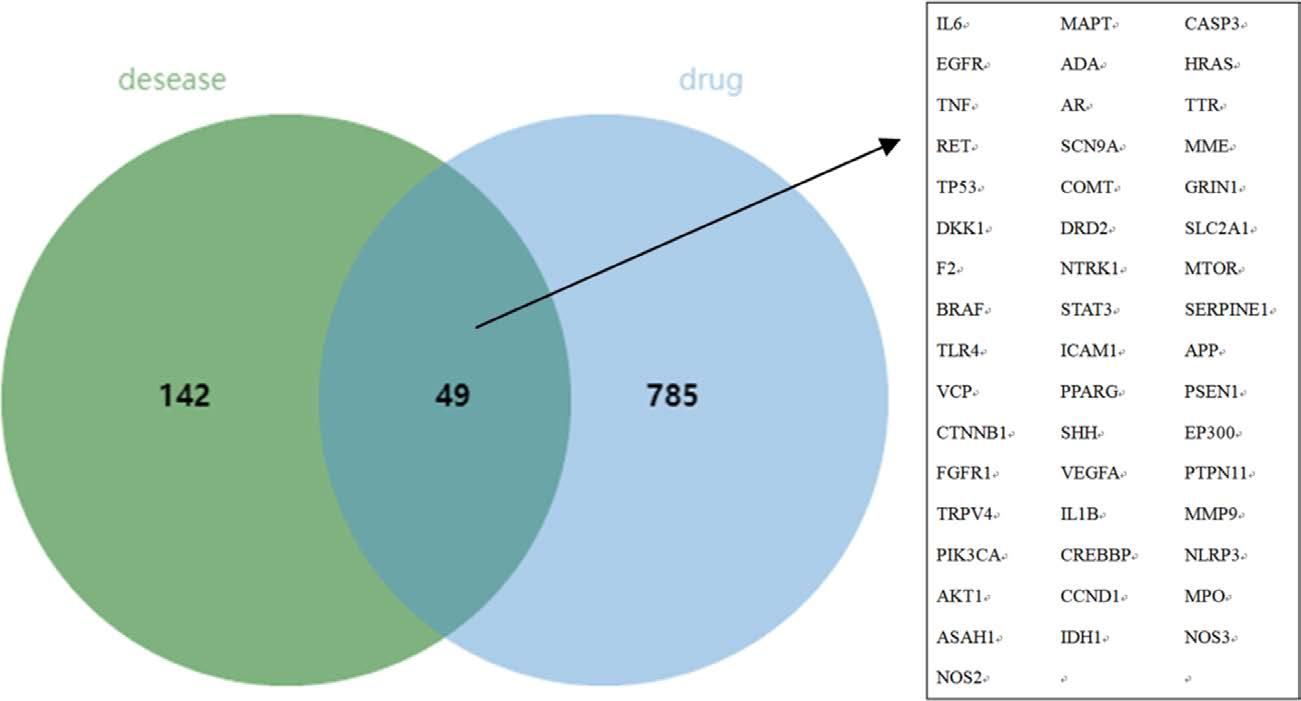
Venn diagram.

The Venn diagram is presented in Figure 2 by the intersection of BYHWD’s action target and the SCI illness target. The green portion represents the SCI target, the blue portion represents the BYHWD target, and the overlapping portion represents the common target. As illustrated in the table, a total of 49 intersecting targets were identified, including IL6, MAPT, CASP3, EGFR, ADA, HRAS, TNF, AR, TTR, RET, SCN9A, MME, TP53, COMT, GRIN1, DKK1, DRD2, SLC2A1, F2, NTRK1, MTOR, BRAF, STAT3, SERPINE1, TLR4, ICAM1, APP, VCP, PPARG, PSEN1, CTNNB1, SHH, EP300, FGFR1, VEGFA, PTPN11, TRPV4, IL1B, MMP9, PIK3CA, CREBBP, NLRP3, AKT1, CCND1, MPO, ASAH1, IDH1, NOS3, NOS2.

### 3.4. Construction and analysis of PPI network

**Figure 3.**
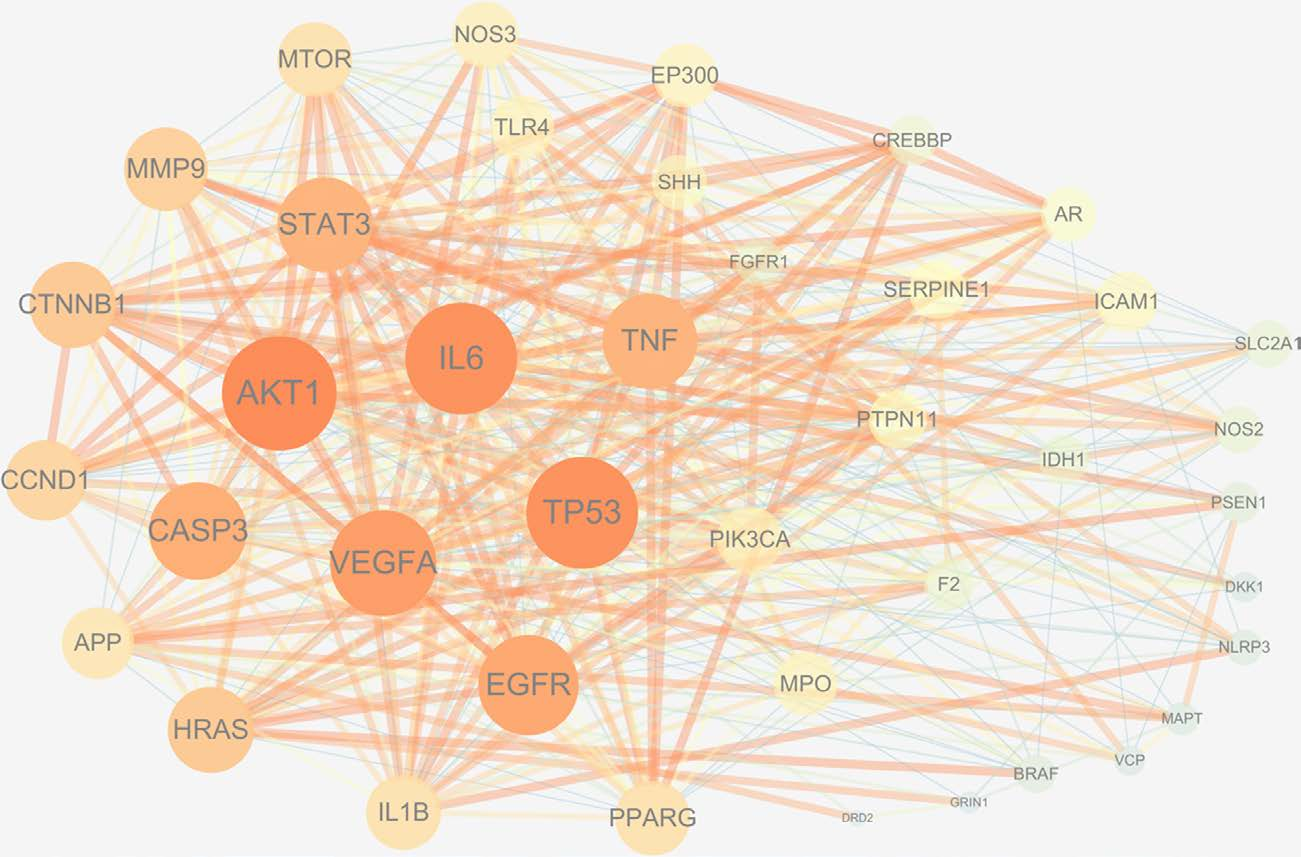
PPI network diagram. PPI = protein–protein interaction.

The top 8 potential targets were identified after twice screening the median of the values greater than or equal to Degree: AKT1, TP53, IL6, VEGFA, EGFR, CASP3, STAT3, and TNF, as shown in Table 5. The PPI network of potential therapeutic targets of BYHWD for SCI was visualized using Cytoscape 3.7.2 software (Fig. 3).

**Table 5:** The Top 8 network topology parameters of the degree value of the PPI network graph.

Gene	Protein name	Degree	BC	CC	TC
AKT1	AKTSerine/ThreonineKinase1	39	0.096052	0.842105	0.439637
TP53	TumorProteinP53	38	0.039903	0.813559	0.464726
IL6	Interleukin6	38	0.082867	0.813559	0.44065
VEGFA	VascularEndothelialGrowthFactorA	36	0.029326	0.786885	0.479314
EGFR	EpidermalGrowthFactorReceptor	34	0.026021	0.761905	0.48373
CASP3	Caspase3	33	0.023723	0.75	0.49323
STAT3	SignalTransducerAndActivatorOfTranscription3	32	0.017903	0.738462	0.50133
TNF	TNFReceptorSuperfamilyMember1A	32	0.035114	0.738462	0.484043

AKT1 = recombinant Human Protein Kinase, BP = biological processes, CASP3 = Apoptosis-Related Cysteine Peptidase, CC = closeness centrality, EGFR = epidermal growth factor receptor, IL6 = interleukin-6, PPI = protein-protein interaction, STAT3 = signal transducer and activator of transcription 3, TC = topological coefficient, TNF = Tumor Necrosis Factor, TP53 = Tumor Protein p53, VEGFA = vascular endothelial growth factor.

**Figure 4.**
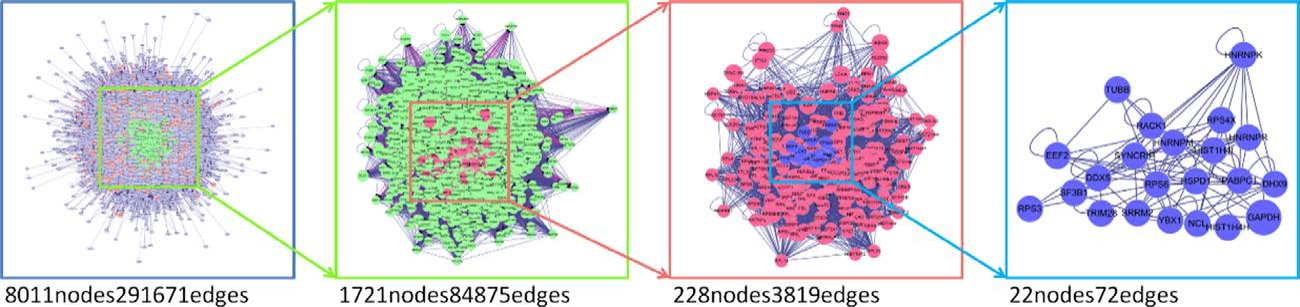
Topological analysis of the intersection of Buyang Huanwu Decoction and SCI. SCI = spinal cord injury.

In order to determine the top 10 core targets of Degree value, the intersection of the active ingredient targets of Tonic Yang Returning Five Soup and SCI disease targets were taken and visualized by Cytoscape 3.7.2 software (Fig. 4). The top 10 targets were HNRNPM, NCL, HNRNPK, DDX5, TUBB, HIST1H4I, SYNCRIP, HIST1H4H, DHX9, and PABPC, as detailed in Table 6.

**Table 6:** Top 10 core targets by degree value in topological analysis.

Name	Betweenness	Closeness	Degree	LAC	NeighborhoodConnectivity
HNRNPM	5240.247	0.534327	274	40.95946	220.4051
NCL	4635.284	0.532178	273	38.1831	233.738
HNRNPK	4517.764	0.532508	270	40.86385	239.8993
DDX5	5495.832	0.533333	269	33.26484	254.9064
TUBB	4002.464	0.530046	262	31.62626	227.8855
HIST1H4I	3114.974	0.528418	260	28.64737	221.6115
SYNCRIP	3664.612	0.526155	259	29.69364	218.5907
HIST1H4H	2346.865	0.526799	252	29.01105	215.9246
DHX9	3838.661	0.528905	251	39.08543	233.0201
PABPC1	3687.905	0.528905	243	41.45128	226.9544

### 3.5. GO and KEGG analysis

**Figure 5.**
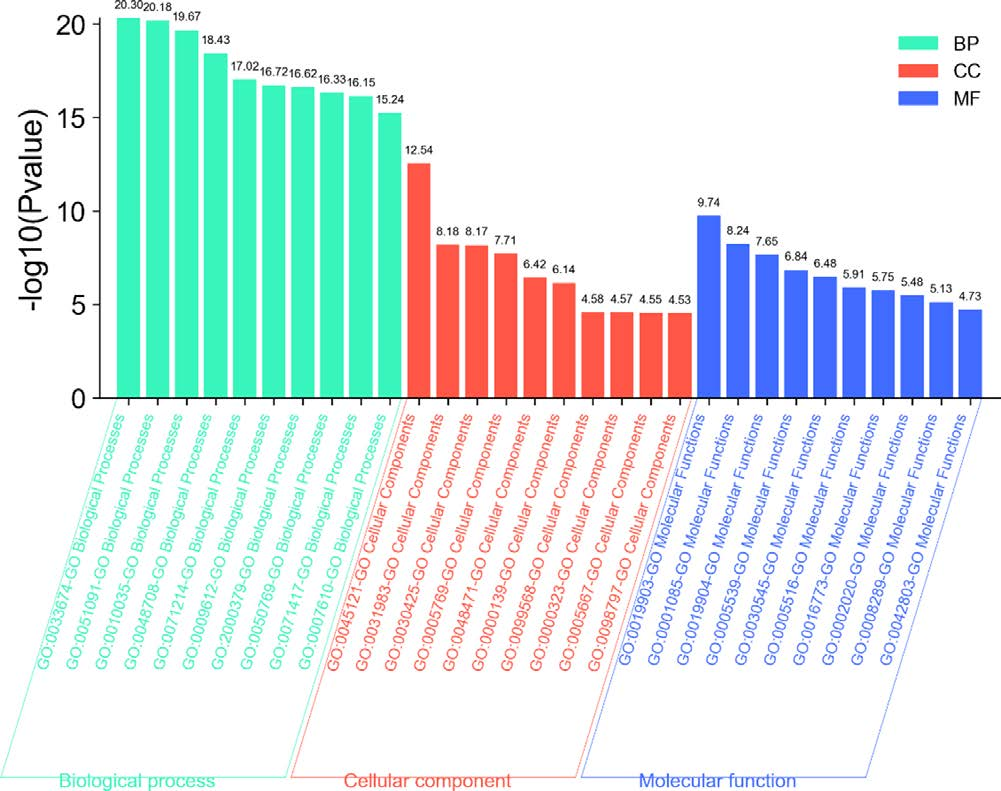
Bar graph of GO enrichment analysis results. BP = biological processes, CC = closeness centrality, GO = gene ontology, MF = molecular function.

Figure 5 displays a bar graph showing the findings from the GO enrichment study, and Table 7 contains the findings from the GO analysis: There are 1492 enrichment findings for the biological process, and after hierarchical clustering, the pathway GO:0033674, which enriches 20 genes, has the lowest *P* value. This pathway is followed by GO:0051091, which enriches 16 genes. Positive control of kinase activity, positive regulation of DNA-binding transcription factor activity, and regulation of inorganic substance response were the key functions of the top-ranked genes. After hierarchical clustering, the pathway GO:0045121 in the cellular fraction, which had a total of 74 enrichment findings, had the lowest *P* value and enriched 12 genes, followed by GO:0031983, which enriched 9 genes. The top ranking mostly involves (nerve direction) membrane rafts, sacs, and dendrites. There are a total of 73 enrichment findings in the molecular functions. GO:0019903 has the lowest *P* value after hierarchical clustering, enriching 8 genes, followed by GO:0001085, enriching 7 genes. Protein phosphatase binding, RNA polymerase II transcription factor binding, and protein domain specific binding are among the top ranks.

**Table 7:** GO-related genes.

Path name	Related genes	log *P* value	Number of genes
GOBP:0033674-positiveregulationofkinaseacti vity (positive regulation of kinase activity)	AKT 1, CCND 1, BRAF, DRD2, EGFR, F2, FGFR1, MTOR, HRAS, IL1B, MAPT, NTRK1, PIK3CA, PSEN 1, PTPN11, RET, TLR4, TNF, VEGFA, DKK1, APP, AR, ICAM1, IL6,CASP3,GRIN1,MMP9,STAT3,CREBBP,EP300,TP53,NLRP3	−20.2989	20
GOBP:0051091-positive regulation of DNA-binding transcription factor activity (positive regulation of DNA-binding transcription factor activity)	AKT 1,APP,AR,CTNNB1,EP300,ICAM1,IL1B,IL6,NTRK1,PPARG,SHH,STAT3,TLR4,TNF,VEGFA,NLRP3,ADA,MTOR,MMP9,PSEN1,TP53,CASP3,DRD2,HRAS,NOS3	−20.183	16
GOBP:0010035-responsetoinorganicsubstance (response to inorganic substances)	ADA,AKT 1,APP,CCND1,BRAF,CASP3,DRD2,EGFR,ICAM1,IL6,MAPT,MMP9,MPO,NOS3,NTRK1,PIK3CA,SHH,TNF,VCP	−19.667	19
GOBP:0048708-astrocytedifferentiation	APP,EGFR,F2,IL6,MAPT,PSEN1,PTPN11,SHH,STAT3,TLR4,TNF,AKT1,MTOR,PPARG,IL1B,CREBBP,CTNNB1,DRD2,EP300,HRAS,SERPINE1,NLRP3,CCND1,MMP9,TP53, VEGFA, DKK1, TRPV4, NOS2, AR, ICAM1, NTRK1, ADA, FGFR1, MPO	−18.4275	11
GOBP:0071214-cellularresponsetoabioticstimulus (cellular responses to abiotic stimuli)	AKT 1, CASP 3, CREBBP, EGFR, EP300, HRAS, IL1B, MME, MMP9, PIK3CA, PTPN11, SLC2A1, TLR4, TP53, TRPV4, APP, CCND1, DRD2, MTOR, GRIN1, ICAM1, NTRK1	−17.0184	15
GOBP:0009612-responsetomechanicalstimulus (mechanical stimulus response)	AKT 1, CTNNB 1, DRD2, EGFR, IL1B, MPO, NTRK1, PIK3CA, PPARG, PTPN11, SLC2A1, TLR4, TRPV4, MTOR, ICAM1, TP53, STAT3, IL6, AR, EP300, SHH	−16.7189	13
GOBP:2000379-positiveregulationofreactiveoxygenspeciesmetabolicprocess	AKT 1,EGFR,F2,MTOR,GRIN1,ICAM1,IL1B,MAPT,TLR4,TNF,TP53,MPO,NOS2,NOS3,AR,CASP3,EP300,FGFR1,HRAS,MMP9,SERPINE1,PSEN1,RET,SHH,IL6,NLRP3,PPARG,COMT,DRD2,BRAF,PTPN11,SLC2A1	−16.6247	11
GOBP:0050769-positiveregulationofneurogenesis (positive regulation of neurogenesis)	CTNNB 1, DRD 2, EP300, FGFR1, MTOR, IL1B, IL6, MAPT, MME, NTRK1, PPARG, PSEN1, RET, SHH, TNF, VEGFA, NOS3	−16.3266	16
GOBP:0071417-cellularresponsetoorganonitrogencompound (cellular response to organic nitrogen compounds)	AKT 1,APP,CASP3,CTNNB1,MTOR,GRIN1,ICAM1,IL1B,NTRK1,PIK3CA,PPARG,PSEN1,PTPN11,STAT3,TLR4,TNF,TP53,MMP9,SLC2A1,HRAS	−16.1496	17
GOBP:0007610-behavior (behavior)	ADA,APP,CASP3,DRD2,EGFR,EP300,MTOR,GRIN1,MAPT,MME,NTRK1,PSEN1,SCN9A,STAT3,TP53,DKK1,TNF,HRAS,IL1B,AKT1,VCP,BRAF	−15.2434	16
GOCC: 0045121-membraneraft (membrane raft)	APP, CASP 3, EGFR, ICAM1, MAPT, MME, NOS3, PSEN1, RET, SHH, SLC2A1, TNF	−12.5371	12
GOCC: 0031983-vesiclelumen (vesicle)	ADA,APP,EGFR,IDH1,MPO,SERPINE1,TTR,VCP,VEGFA	−8.17746	9
GOCC:0030425-dendrite (dendrite)	ADA,APP,COMT,DRD2,MTOR,GRIN1,MAPT,MME,NTRK1,PSEN1,RET,SCN9A,TRPV4,CASP3,SLC2A1,AKT1	−8.17229	11
GOCC:0005769-earlyendosome (early endosome)	APP, ASAH 1, EGFR, MME, NTRK1, PSEN1, RET, TLR4, DKK1, FGFR1, GRIN1, IL6, PPARG	−7.71136	9
GOCC:0048471-perinuclearregionofcytoplasm (perinuclear region of cytoplasm)	APP, CTNNB 1, EGFR, HRAS, NOS2, PIK3CA, PPARG, PSEN1, TLR4, VCP	−6.42261	10
GOCC:0000139-Golgimembrane (Golgi Membrane)	APP, DRD 2, EGFR, MTOR, HRAS, NOS3, NTRK1, PSEN1, SLC2A1, NLRP3, MPO, CCND1	−6.13667	10
GOCC:0099568-cytoplasmicregion	ADA, CTNNB 1, MAPT, NOS2, PSEN1, SLC2A1, TRPV4, APP, EGFR	−4.58327	7
GOCC:0000323-lyticvacuole (dissolving vacuole)	ADA,ASAH1,MTOR,IL1B,MPO,PSEN1,TTR,VCP	−4.56545	8
GOCC:0005667-transc riptionfactorcomplex	CREBBP, CTNNB 1, EP300, PPAR G, STAT3, TP53	−4.54877	6
GOCC:0098797-plasmamembraneproteincomplex (plasma membrane protein complex)	CASP 3, CTNNB 1, EGFR, GRIN1, IL6, PSEN1, RET, SCN9A	−4.53493	8
GOMF:0019903-proteinphosphatasebinding	AKT 1, CTNNB 1, EGFR, MAPT, PPARG, STAT3, TP53, VCP, CCND1, MTOR, NTRK1, PTPN11, SLC2A1, TRPV4	−9.73956	8
GOMF:0001085-RNApolymeraseIItranscriptionfactorbinding	AR,CREBBP,CTNNB1,EP300,PPARG,STAT3,TP53,CCND1,MTOR,NLRP3,EGFR,MPO,PSEN1,PTPN11,HRAS	−8.24381	7
GOMF:0019904-proteindomainspecificbinding	APP,AR,MTOR,IL1B,MAPT,PPARG,PSEN1,PTPN11,TP53,VCP,TRPV4	−7.65201	11
GOMF:0005539-glycosaminoglycanbinding	APP,F2,FGFR1,MPO,SHH,VEGFA,NLRP3	−6.8449	7
GOMF:0030545-receptorregulatoractivity	APP, F2, IL1B, IL6, SHH, TNF, TTR, VEGFA, DKK1, PSEN1, CASP3, NTRK1	−6.4776	9
GOMF:0005516-calmodulinbinding	AKT 1,EGFR,GRIN1,NOS2,NOS3,TRPV4,IDH1,PPARG,MPO	−5.91203	6
GOMF:0016773-phosphotransferaseactivity,alcoholgroupasacceptor(phosphotransferase activity, alcohol group as acceptor)	AKT 1, CCND 1, BRAF, EGFR, FGFR1, MTOR, NTRK1, PIK3CA, RET	−5.74733	9
GOMF:0002020-proteasebinding	CASP 3, SERPINE 1, TNF, TP53, VCP, APP	−5.48413	5
GOMF:0008289-lipidbinding	,AR,F2, MAPT,MME,PPARG,TLR4,VCP,TRPV4,GRIN1	−5.1262	9
GOMF:0042803-proteinhomodimerizationactivity	AKT 1, FGFR 1, IDH1, MME, NOS2, NTRK1, STAT3, VEGFA	−4.73268	8

GO = Gene ontology.

The top ranking mostly involves (nerve direction) membrane rafts, sacs, and dendrites. There are a total of 73 enrichment findings in the molecular functions. GO:0019903 has the lowest *P* value after hierarchical clustering, enriching 8 genes, followed by GO:0001085, enriching 7 genes. Protein phosphatase binding, RNA polymerase II transcription factor binding, and protein domain specific binding are among the top ranks.

**Figure 6.**
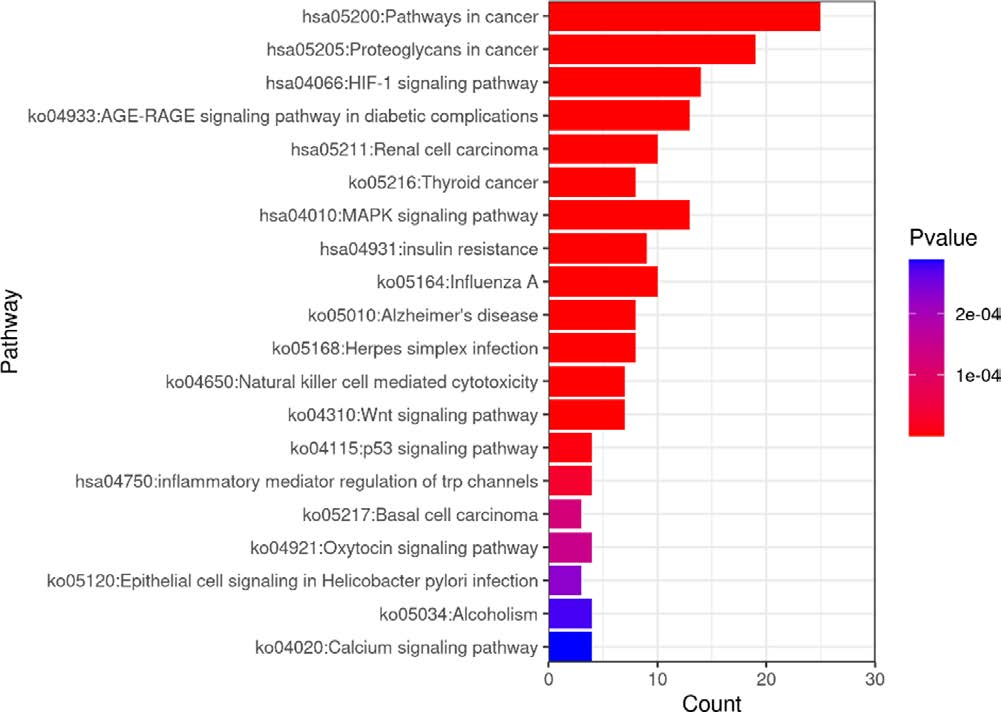
Bar graph of 20 KEGG pathway Enrichment before Clustering (Count value of bar graph represents Hitgenelist). KEGG = Kyoto encyclopedia of genes and genomes.

**Figure 7.**
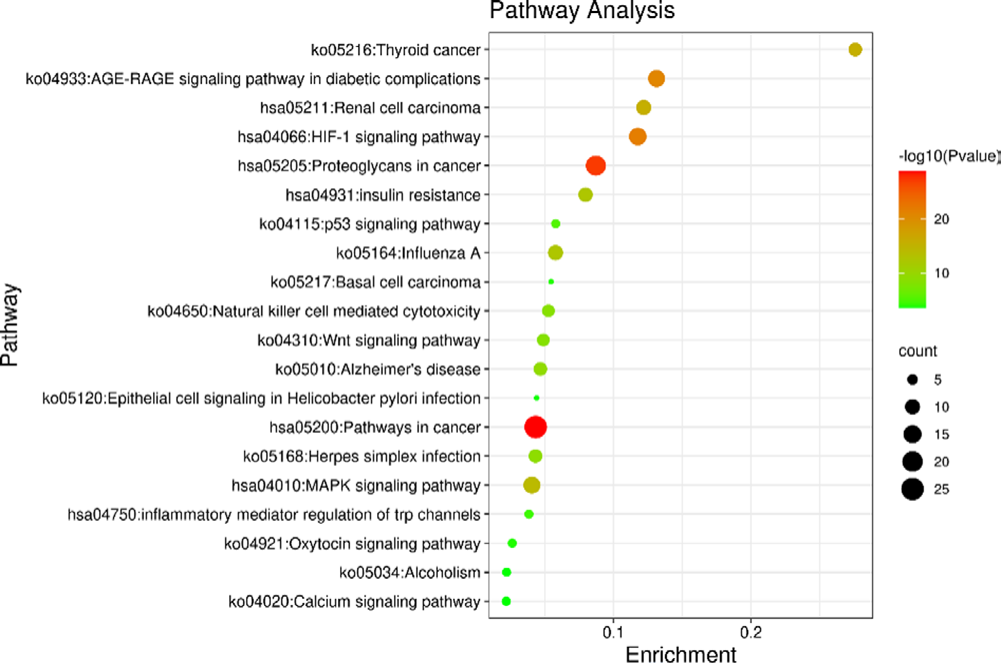
Bubble map of 20 KEGG Pathway Enrichment before Clustering (Enrichment of bubble map stands for Hitgenelist/GeneInGO)). KEGG = Kyoto encyclopedia of genes and genomes.

The results of KEGG analysis showed that 49 targets of Buyang Huanwu Decoction in the treatment of SCI were enriched in 243 channels (see Table 8). See Figures 6 and 7 for the enrichment bar and bubble charts of the top 20 KEGG pathways.

**Table 8:** KEGG-related genes.

Path name	Related genes	log *P* value	Number of genes
hsa05200:Pathwaysincancer	AKT 1, AR, CCND1, BRAF, CASP3, CREBBP, CTNNB1, EGFR, EP300, F2, FGFR1, MTOR, HRAS, IL6, MMP9, NOS2, NTRK1, PIK3CA, PPARG, RET, SHH, SLC2A1, STAT3, TP53, VEGFA	−28.84227728	25
hsa05205: Proteoglycansincancer	AKT 1, CCND 1, BRAF, CASP3, CTNNB1, EGFR, FGFR1, MTOR, HRAS, IL6, MMP9, PIK3CA, PTPN11, SHH, STAT3, TLR4, TNF, TP53, VEGFA, AR, CREBBP, EP300, IDH1, NTRK1, RET, SLC2A1, ICAM1, IL1B, PSEN1, NOS3, DRD2, GRIN1, MPO, SERPINE1, TRPV4, NOS2, F2, NLRP3, ASAH1, PPARG	−27.26509884	19
hsa04066:HIF-1signalingpathway(HIF-1signaling pathway)	AKT 1,CREBBP,EGFR,EP300,MTOR,IL6,NOS2,NOS3,SERPINE1,PIK3CA,SLC2A1,STAT3,TLR4,VEGFA	−21.79901264	14
ko04933:AGE-RAGEsignalingpathwayindiabeticcomplications	AKT 1, CCND 1, CASP3, HRAS, ICAM1, IL1B, IL6, NOS3, SERPINE1, PIK3CA, STAT3, TNF, VEGFA, CTNNB1, MMP9, TP53, TRPV4	−20.87809044	13
hsa05211: Renalcellcarcinoma (renal cell carcinoma)	AKT 1, BRAF, CREBBP, EP300, HRAS, IL6, PIK3CA, PTPN11, SLC2A1, VEGFA, DRD2, GRIN1	−15.72615493	10
ko05216: Thyroidcancer	CCND 1, BRAF, CTNNB1, HRAS, NTRK1, PPARG, RET, TP53, IL6, MMP9, MPO	−15.71277325	8
hsa04010: MAPKsignalingpathway (MAPK signaling pathway)	AKT 1,BRAF,CASP3,EGFR,FGFR1,HRAS,IL1B,IL6,MAPT,NTRK1,TNF,TP53,VEGFA,F2,PIK3CA	−14.11888129	13
hsa04931:insulinresistance (insulin resistance)	AKT 1, MTOR, IL6, NOS3, PIK3CA, PTPN11, SLC2A1, STAT3, TNF	−12.45186039	9
ko05164: InfluenzaA (Influenza A)	AKT 1,CREBBP,EP300,ICAM1,IL1B,IL6,PIK3CA,TLR4,TNF,NLRP3,NOS2,SERPINE1,HRAS,PTPN11,CASP3,MMP9,VCP,VEGFA,STAT3,PPARG,MME,MTOR,EGFR,TTR	−12.40080111	10
ko05010: Alzheimer’s disease	APP, CASP 3, GRIN1, IL1B, MAPT, MME, PSEN1, TNF	−9.239252345	8
ko05168: Herpes simplexinfection (herpes simplex virus infection)	CASP 3,CREBBP,EP300,IL1B,IL6,PTPN11,TNF,TP53,AKT1,MTOR,PIK3CA,GRIN1,PPARG	−8.968213939	8
ko04650: Naturalkillercellmediatedcytotoxicity	BRAF,CASP3,HRAS,ICAM1,PIK3CA,PTPN11,TNF,CTNNB1,MMP9,APP,SHH	−8.484542141	7
ko04310: Wntsignalingpathway (Wnt signaling pathway)	CCND 1, CREBBP, CTNNB1, EP300, PSEN1, TP53, DKK1, BRAF, GRIN1, HRAS, EGFR, FGFR1, TNF	−8.264885797	7
ko04115: p53signalingpathway	CCND 1, CASP 3, SERPINE1, TP53, BRAF, CTNNB1, EGFR, ICAM1	−5.189836315	4
hsa04750: inflammatory mediatorregulationoftrpchannels	IL1B,NTRK1, PIK3CA,TRPV4	−4.483447663	4
ko05217: Basalcellcarcinoma (basal cell carcinoma)	CTNNB 1,SHH,TP53	−3.912861686	3
ko04921: Oxytocinsignalingpathway	CCND1,EGFR,HRAS,NOS3,DRD2,NTRK1,RET	−3.842755254	4
ko05120: Epithelial cell signaling in Helicobacter pyloriinfection (Epithelial cell signaling in Helicobacter pylori infection)	CASP3,EGFR,PTPN11	−3.638673062	3
ko05034: Alcoholism	BRAF, DRD 2, GRIN1, HRAS, F2, NLRP3	−3.561843212	4
ko04020:Calciumsignalingpathway	EGFR, GRIN 1, NOS2, NOS3	−3.543593826	4

KEGG = Kyoto encyclopedia of genes and genomes.

### 3.6. Molecular docking

**Figure 8.**
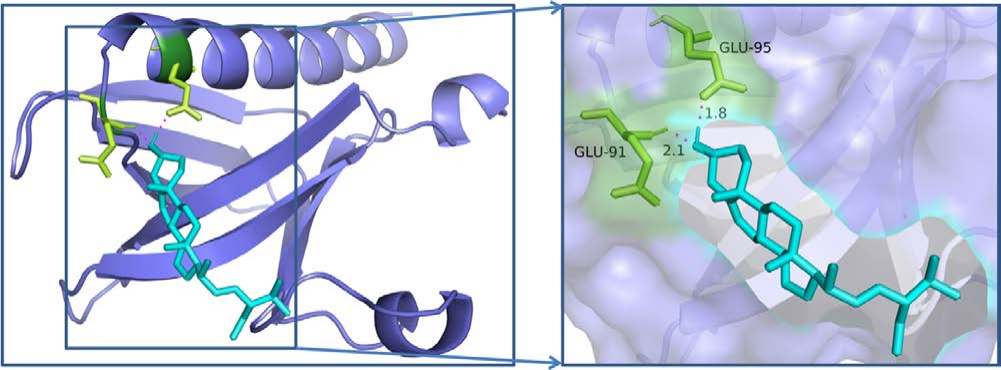
Molecular docking diagram of AKT1 and stigmasterol. AKT1 = recombinant Human Protein Kinase.

**Figure 9.**
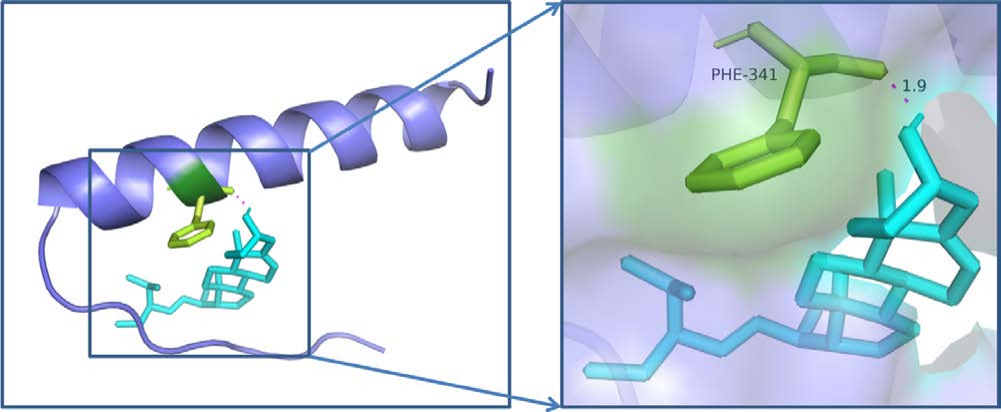
Molecular docking diagram of TP53 and stigmasterol. TP53 = Tumor Protein p53.

**Figure 10.**
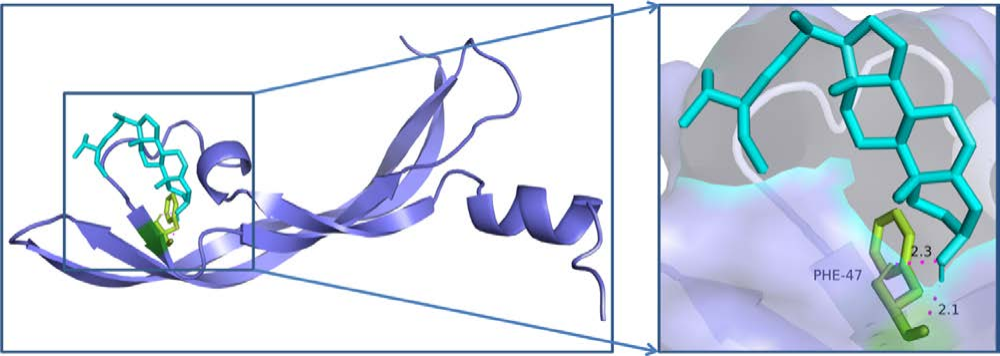
Molecular docking diagram of VEGFA and stigmasterol. VEGFA = vascular endothelial growth factor.

The key chemicals Hederagenin, Quercetin, Baicalein, Beta-Sitosterol, and Stigmasterol were chosen to dock with the PPI network’s core targets, AKT1, TP53, IL6, VEGFA, EGFR, CASP3, STAT3, and TNF, and the results were promising. Table 9 shows that if the binding energy is less than zero, the ligand molecule can spontaneously bind to the target protein, and the lower the binding energy, the more stable the molecule binds to the target protein. In the AutoDock context, a binding energy absolute value larger than 5 suggests better binding. For graphical analysis, the docking conformations of AKT1 and Stigmasterol, TP53 and Stigmasterol, and VEGFA and Stigmasterol were chosen Figures 8–10.

**Table 9:** Docking binding energies of Buyang Huanwu Decoction core compounds and core targets.

Target protein	Binding free energy/(kJ/mol)				
	Hederagenin	Quercetin	Baicalein	Beta-Sitosterol	Stigmasterol
AKT1	−6.81	−5.43	−6.46	−7.69	−8.64
TP53	−7.38	−5.86	−6.15	−8.09	−8.53
IL6	−7.12	−5.52	−6.5	−7.3	−7.41
VEGFA	−6.73	−5.83	−6.38	−7.66	−7.83
EGFR	−5.43	−3.82	−4.63	−6.25	−5.7
CASP3	−7.39	−5.87	−5.6	−7.0	−7.51
STAT3	−6.56	−4.23	−4.46	−5.76	−6.35
TNF	−6.64	−5.43	−5.16	−6.69	−6.44

AKT1 = recombinant Human Protein Kinase, CASP3 = Apoptosis-Related Cysteine Peptidase, EGFR = epidermal growth factor receptor, IL6 = interleukin-6, STAT3 = signal transducer and activator of transcription 3, TNF = tumor necrosis factor, TP53 = tumor protein p53, VEGFA = vascular endothelial growth factor.

## 4. Discussion

SCI is a widespread, devastating condition of the central nervous system that places a heavy financial burden on the whole community in addition to seriously harming sufferers’ physical and emotional health.^[19]^ We must better investigate the pathophysiology of SCI and find novel therapy approaches in order to solve the treatment conundrum. Neuroprotection and regeneration are now the major methods for treating spinal cord injuries.^[20]^ However these therapies, particularly the later regenerative repair procedure, have not yet had satisfactory results.^[9]^ TCM, which originated in China, has had success in treating certain illnesses.^[21]^ According to TCM, the injury to the Governor Vessel, the stoppage of qi and blood circulation, the loss of nutrition to the bones, muscles, and bones, atrophy of the limbs, and stasis of qi and blood are the basic etiologies of this disease.^[22]^ BYHWD, a decoction that is frequently used in TCM, has been shown to have a considerable therapeutic impact in the treatment of SCI. Its actions include tonifying qi, stimulating blood, cleansing meridians, and activating collaterals.^[23]^ However, the specific mechanism of the compound in the treatment of SCI needs to be further elucidated.

This investigation into the mechanism of action of BYHWD in the treatment of SCI was based on network pharmacology methodology. The BYHWD was found to have 109 compounds and 98 active ingredients, among which Hederagenin, Quercetin, Cortexin, Baicalein, Beta-Sitosterol, and Stigmasterol may be crucial elements in the therapy of SCI. Helexin has pharmacological actions that include anti-inflammatory, liver protection, anticoagulant, antidepressant, anticancer, antibacterial, anti-AS, and others.^[24]^ Additionally, it can increase the expression of Bax while decreasing the expression of Bcl-2, which would eventually encourage cell death.^[25]^ Quercetin, β-sitosterol, and stigmasterol have anti-inflammatory and antioxidant effects.^[26,27]^ According to studies, quercetin can chelate metal ions, scavenge reactive oxygen radicals, and prevent oxidative damage to low-density lipoproteins. The production of inflammatory cytokines and the activity of inflammatory enzymes are both inhibited at the same time.^[28]^ In order to minimize the synthesis of inflammatory factors and TLR4 overexpression, β-sitosterol can block the activation of NLRP3, the activation of the MAPK signaling pathway, and the activation of NLRP3.^[29]^

Through network analysis, it was shown that AKT1, IL6, TP53, VEGFA, EGFR, CASP3, TNF, STAT3, CTNNB1, and HRAS may be the primary targets of BYHWD in the treatment of SCI. Three members of the serine/threonine protein kinase family, known as AKT (AKT1, AKT2, and AKT3), are closely connected to one another and are involved in a number of biological activities. The PI3K/Akt system is strongly associated with both pro-inflammatory and anti-inflammatory responses, and the Akt signaling pathway has the ability to regulate activities including inflammatory cytokines and macrophage phagocytosis.^[30,31]^ An essential tumor suppressor gene called TP53 is involved in cell cycle arrest, apoptosis, senescence, and carcinogenesis.^[32]^ MicroRNAs (miRNAs) are a part of the TP53 signaling pathway. According to research, miR-429-5p controls the expression of Bcl-2, which controls the apoptosis of spinal cord cells and contributes to the healing process after SCI.^[33]^ The caspase family, which is classified into apoptosis-related caspases and inflammation-related caspases, is a collection of highly homologous and structurally identical proteases. Specifically, caspase3 controls apoptosis.^[34]^ According to studies, BYHWD can help rats recover their neurological function after a SCI by suppressing the caspase cells’ apoptotic process.^[35]^ The STAT protein family, which includes STAT3, is activated by phosphorylation in response to a number of cytokines and growth factors.^[36,37]^ The JAK2/STAT3 signaling system controls the production of several cytokines and growth factors as well as cell proliferation, differentiation, and death. It is also linked to the incidence and progression of acute SCI. We propose that the inflammatory response, cellular autophagy, and the apoptotic process can control neurological impairment and recovery in tonic yang and rejuvenation soup.^[38]^

BYHWD can also cure SCI by controlling inflammation, antioxidant stress, and apoptosis via the cancer route, HIF-1 signaling pathway, MAPK signaling pathway, and Wnt signaling pathway, among other pathways, according to the KEGG enrichment study. We discovered that BYHWD can help neural stem cell transplantation, which is mostly controlled by wnt signaling, to cure spinal cord damage in experimental rats, boost the growth of mitotic cells in the spinal cord, and thereafter, to some extent, support the restoration of neurological function. Additionally, several researchers have discovered that BYHWD can lessen secondary damage to the spinal cord brought on by ischemia and hypoxia, ameliorate ischemia and hypoxia, and stimulate the production of HIF-1 and VEGF in the SCI section of SD rats following SCI. support the restoration of nerve function after sexual damage.^[35]^ More research is required to more conclusively demonstrate the mechanism of action of BYHWD, as there are currently only a small number of studies on its use in the treatment of SCI.

## 5. Conclusion

BYHWD, a tonic, possesses anti-inflammatory and antioxidant properties, suppresses apoptosis, and aids in the healing of nerve cells. Several targets that are important for treating SCI can be affected by a single chemical, and each target may be connected to a number of pathways. Clinically, BYHWD is successful in treating SCI, although it’s unknown how it works. Through the use of TCM network pharmacology and molecular docking tools, we investigated the mechanism of multi-component, multi-target, and multi-channel therapy of SCI in this research. It draws attention to the legitimacy and efficacy of BYHWD, which offers clinical practitioners direction in the treatment of SCI and fresh thoughts for further examining the mechanism of action of BYHWD.

## Acknowledgments

The authors would like to thank the Sichuan Vocational College of Health and Rehabilitation for helpful discussions on topics relevant to this work.

## Author contributions

**Conceptualization:** Lan Chen.

**Data curation:** Ying Wang.

**Formal analysis:** Ying Wang and Lan Chen.

**Investigation:** Hai-Xu Chen.

**Methodology:** Ying Wang and Hai-Xu Chen.

**Project administration:** Lan Chen, Ying Wang, and Hai-Xu Chen.

**Resources:** Jun-Wei Wang, Xin Chen.

**Software:** Jun-Wei Wang.

**Supervision:** Ying Wang and Lan Chen.

**Validation:** Jun-Wei Wang.

**Writing – original draft:** Ying Wang.

**Writing – review & editing:** Lan Chen.
